# State of the art forensic techniques reveal evidence of interpersonal violence *ca*. 30,000 years ago

**DOI:** 10.1371/journal.pone.0216718

**Published:** 2019-07-03

**Authors:** Elena F. Kranioti, Dan Grigorescu, Katerina Harvati

**Affiliations:** 1 Department of Forensic Sciences, Medical School, University of Crete, Heraklion, Greece; 2 Department of Medical Imaging, Heraklion University Hospital, Heraklion, Greece; 3 Edinburgh Unit for Forensic Anthropology, HCA, University of Edinburgh, Edinburgh, United Kingdom; 4 Dept. of Geology, University of Bucharest, Bucharest, Romania; 5 Paleoanthropology, Senckenberg Centre for Human Evolution and Palaeoenvironments, Eberhard Karls Universität Tübingen, Tübingen, Germany; 6 DFG Centre of Advanced Studies ‘Words, Bones, Genes, Tools: Tracking Linguistinc, Cultural and Biological Trajectories of the Human Past’, Eberhard Karls University of Tübingen, Tübingen, Germany; Max Planck Institute for the Science of Human History, GERMANY

## Abstract

The Cioclovina (Romania) calvaria, dated to *ca*. 33 cal ka BP and thought to be associated with the Aurignacian lithic industry, is one of the few relatively well preserved representatives of the earliest modern Europeans. Two large fractures on this specimen have been described as taphonomic modifications. Here we used gross and virtual forensic criteria and experimental simulations on synthetic bone models, to investigate their nature. Both forensic trauma pattern analysis and experimental models exclude a postmortem origin for the Cioclovina fractures. Rather, they indicate two incidents of blunt force trauma, the second clearly inflicted with a club-like object. The magnitude and extent of the lesions and the lack of signs of healing indicate a fatal injury. The Upper Paleolithic period is noted for intensified technological innovation, increased symbolic behavior, and cultural complexity. We show that the behavioural repertoire of the earliest modern Europeans also comprised violent inter-personal interactions and murder.

## Introduction

**Fig 1:**
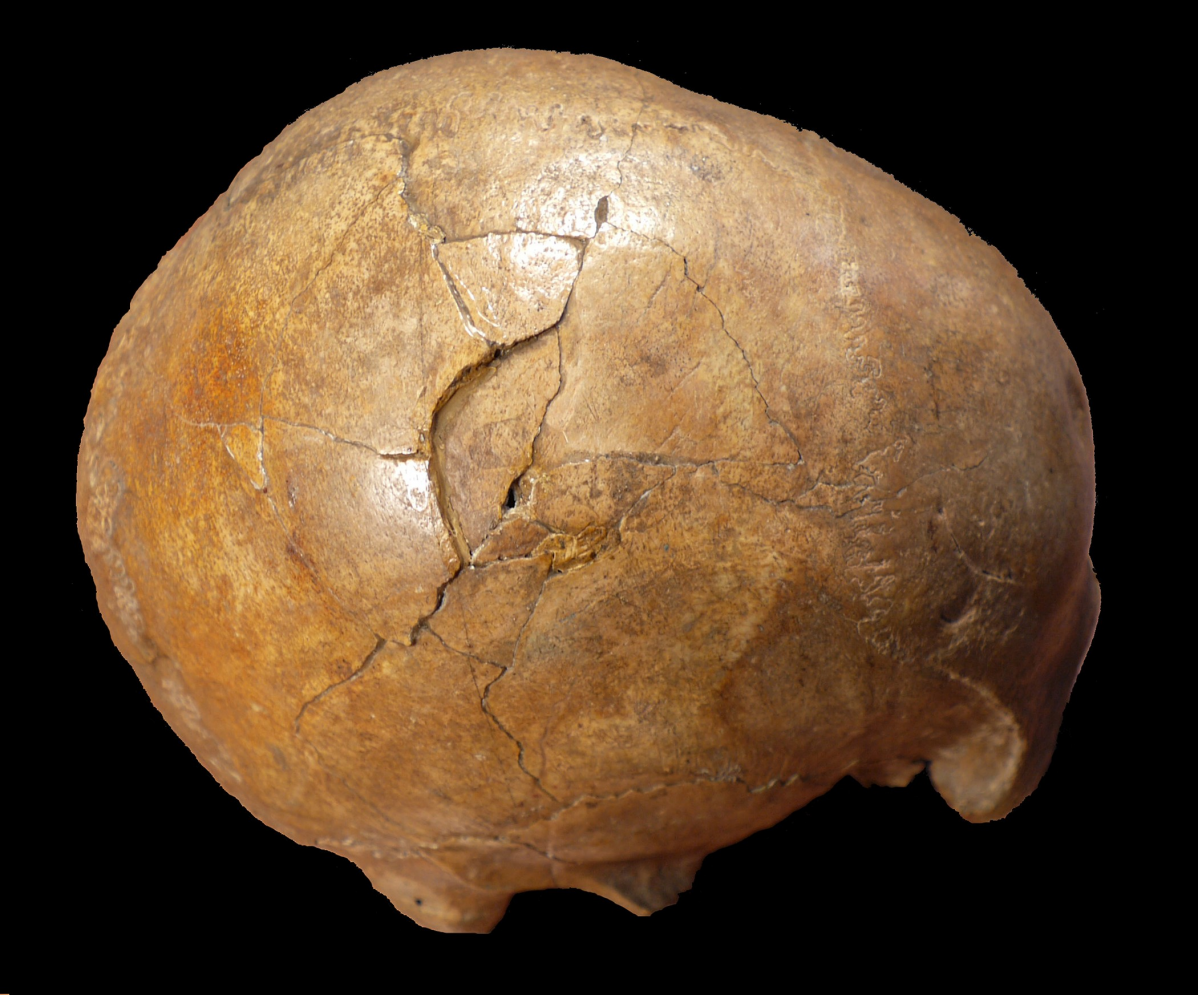
Right lateral view of the Cioclovina calvaria exhibiting a large depressed fracture.

The Cioclovina calvaria (Fig 1) was discovered in 1941 during phosphate mining of the Pestera Cioclovina cave, South Transylvania, together with three possibly Aurignacian stone tools and several cave bear fossils [1–4]. Although its archaeological context is not well understood -as it was not recovered from controlled excavation-, and despite being an isolated calvaria, it is among the earliest directly dated and relatively well preserved, modern humans known from Europe, dating to *ca*. 33 thousand calendar years before present[4]. It is therefore crucial for our understanding of the early modern human populations of the continent. The fossil preserves the vault and most of the cranial base of an adult male individual. Although initially considered female[1,3], its sex was recently confirmed as male through the analysis of the bony labyrinth morphology[5] and of aDNA[6]. Previous descriptions have noted the presence of antemortem trauma in the form of two small, healed scars on the frontal bone[1,2]. The nature of a large fracture on the right parietal and extending to most of the posterior aspect of the cranium, however, is disputed[2,7]. While some authors[7] have considered it the result of perimortem injury, more recent work[2] has questioned this interpretation on the grounds that this fracture was not described in the original 1942 publication[1]. Here we reassess the skeletal trauma of the Cioclovina calvaria by means of visual inspection, Computed Tomography (CT) and experimental trauma analysis, followed by a forensic interpretation of the findings. The results of our study strongly suggest that the Cioclovina fractures represent indisputable hard evidence of fatal interpersonal violence among early Upper Paleolithic modern humans of Europe.

## Materials and methods

### The Cioclovina calvaria

The Cioclovina fossil human calvaria is housed at the Bucharest University Laboratory of Paleontology, Vertebrate Paleontology collection (Catalogue number FGGUB 307), curated until 2017 by one of us (DG). The specimen is accessible to specialists is by request addressed to the person in charge of the Vertebrate Paleontology collection.

### Computed tomography and virtual reconstruction

The Cioclovina calvaria was scanned with a Siemens sensation 64 medical CT scanner at the Centrul De Sanatate Pro-Life SRL, Bucharest[see 4]. Scanning direction was coronal (transverse). Slice thickness of 0.625 mm, X-ray tube voltage 120 kV and tube current 304 mA were used. More details can be found in Kranioti et al.[4] and Uhl et al[5]. CT analysis was performed with the Amira 5.2 and 6.5 and Aviso 6.2 software packages.

### Timing of injuries

Antemortem trauma occurs prior to death and it can be accompanied by signs of healing such as remodeling by callus formation in long bones [8] or creation of bony bridges between fragments in the cranium[9]. Studies on American Civil War victims report osteoclastic activity 5–7 days post-injury [10,11]. Osseous discontinuities lacking signs of remodeling can be attributed to either perimortem injury (the bone still preserves its elastic properties) or postmortem breakage (the bone is dehydrated lacking elastic properties). The differentiation between perimortem and postmortem fractures is based on their distinctive patterns. Perimortem trauma in the cranium typically presents plastic deformation of the bone in the location of impact that can be accompanied by small fragments (bone flakes) still attached to the skull [12–15]. Fracture lines tend to migrate towards structurally weaker areas of the skull, such as those where multiple blood vessels merge and naturally occurring foramina are located [13,16]. They can meet and follow suture lines and continue to the adjacent fragment. In addition, fracture lines terminate in preexisting fractures when the energy is fully absorbed. If the energy exceeds a threshold the oldest fracture may elongate, or a new fracture may start from it [17]. Similarly, fracture lines can terminate in sutures if the energy is absorbed or cross them and continue to the adjacent fragment on the opposite side [18].

Postmortem breakage can be a result of exposure to environmental conditions (scavenging, soil pressure, moisture, etc.). Postmortem fractures present characteristic squared and sharp fracture edges—typically perpendicular to the bone surface -, with rough preponderant texture and irregular preponderant outline [12,13,19]. Such fractures do not follow paths of least resistance and are presented in random patterns. Dry bone can be massively fragmented when a force is applied compared to “fresh” bone which still preserves water and elastic properties and is more likely to first bend before braking [20]. Soil pressure can provoke shape changes and result in linear cracks resembling perimortem linear fractures but these have distinctive appearance; they appear dry, may have different (lighter) color from the bone surface and follow the grain of the bone [21].

The timing of injuries (ante-, peri- or post-mortem) was evaluated according to standard forensic criteria [10,12,15] summarized in **Table 1.** These include absence of signs of remodeling or healing, plastic deformation, bone flakes on the impact site, fracture angle and edge morphology and preponderant outline.

**Table 1:** Forensic criteria for estimating the timing of the injuries.

Feature	*Perimortem	*Postmortem
Signs of plastic response: Permanent deformation of the bone after exceeding the elastic response limit.	**Present**	Absent
Bone flakes: Small bone fragments attached to the impact site	**Present**	Absent
Edge morphology: The relative sharpness of the fracture margin	**Sharp, incomplete or bend-edges**	Squared edges at right angles to the bone surface-no bending
Fracture angle: Angle between the cortical table and the direction of the fracture	**Acute or obtuse**	Right
Preponderant outline	**Regular**	Irregular
Cranial bone remodeling: Bony bridges between the fragments	**Absent**	**Absent**
Migration of fracture towards structures of least resistance	**Present: radiating fractures following the canal of a branch of the posterior middle meningeal vessel**	Absent

*Bold descriptions correspond to Cioclovina

### Pattern of injuries

Cranial blunt force trauma (hereafter BFT) can result in linear, depressed or penetrating trauma depending on intrinsic (bone morphology, bone thickness, overlying soft tissue thickness, cortical density, position of the body etc.) and extrinsic (velocity and duration of impact, object shape, weight etc.) factors[12,14,15]. When a force (stress) acts on a bone, it causes bending of the bone (elastic deformation) until it reaches its elastic limit and then fails (plastic deformation), resulting in a permanent fracture. Slow impact allows enough time for the bone to absorb the kinetic energy of the impact and to bend before failure; thus slow velocity BFT is highly correlated with plastic deformation[12,14].

Linear fractures appear more frequently than any other type of fracture and they can have multiple etiology. Some authors have suggested that they are more often recorded in accidents, such as falls, contrary to depressed fractures, which are highly associated with assaults and violent incidents [21,22]. BFT caused by a fall on a solid surface would result in one or more linear fractures radiating from the impact site that, depending on the height of the fall, can be comminuted fractures driving the fragments inwards to the skull [22]. On the other hand, BTF caused by a round-edged instrument, such as a club or a baseball bat, exhibits the following characteristics[12,14,23]:

1. Fracture formation at the impact point due to initial inbending of the cranial vault with peripheral outbending;
2. Inward displacement of the bony fragment due to plastic deformation; Small fragments remaining in place suggest that the impact took place while soft tissue was present.
3. The presence of radiating fractures in the area of outbending, which start at one or more points distant to the impact site, progressing both towards it and in the opposite direction;
4. The presence of concentric fractures forming perpendicular to the radiating fractures.

For the differentiation of the two types of cranial BFT, the Hat Brim Line (HBL) Rule is proposed in many forensic pathology handbooks[24]. The HBL corresponds to the maximum circumference of the vault, and lesions above it are more frequent in blow rather than in fall injuries. Fracaso and colleagues[25] add that blunt force injuries from falls lie below the HBL if the following conditions are fulfilled a) Body in standing position before fall b) Fall from one’s height c) Flat floor d) Absence of intermediate obstacles.

### Number and sequence of injuries

The estimation of the number of injuries and the time sequence in which they occurred followed a standard criterion known also as Puppe’s Rule[26]: a fracture track from a second blow will not cross a previous fracture. It is widely accepted that multiple, rather than single, cranial injuries are more typically associated with assault.

### Trauma simulation with artificial bone spheres (SYNBONE)

**Twelve** synthetic bone spheres (7 mm thick) purchased from SYNBONE were used in the experimental process. The hollow spheres comprise two hemi-spheres of modified cranial-like polyurethane glued together designed for ballistics testing. The spheres are filled with 10% solution of GELITA type 3 ballistic gelatin mixed with 360 grams (gm) of ballistic powder to 3.2 l of water per sphere and chilled at 4 C for twenty-four hours, to represent the brain. Six different scenarios were tested twice with blunt force trauma simulations in SYNBONE spheres. These included:

1. Fall from height
2. Single blow with a rock
3. Single blow with a bat like object on the vault head free to move

1. Single blow with a bat like object near the foramen magnum head free to move
2. Two blows to the head with a baseball bat head free to move
3. Two blows to the head with a baseball bat head against a solid surface

Information on the experimental procedure can be found in the Supplementary 1 Online Material (S1 Online Material).

## Results

**Fig 2:**
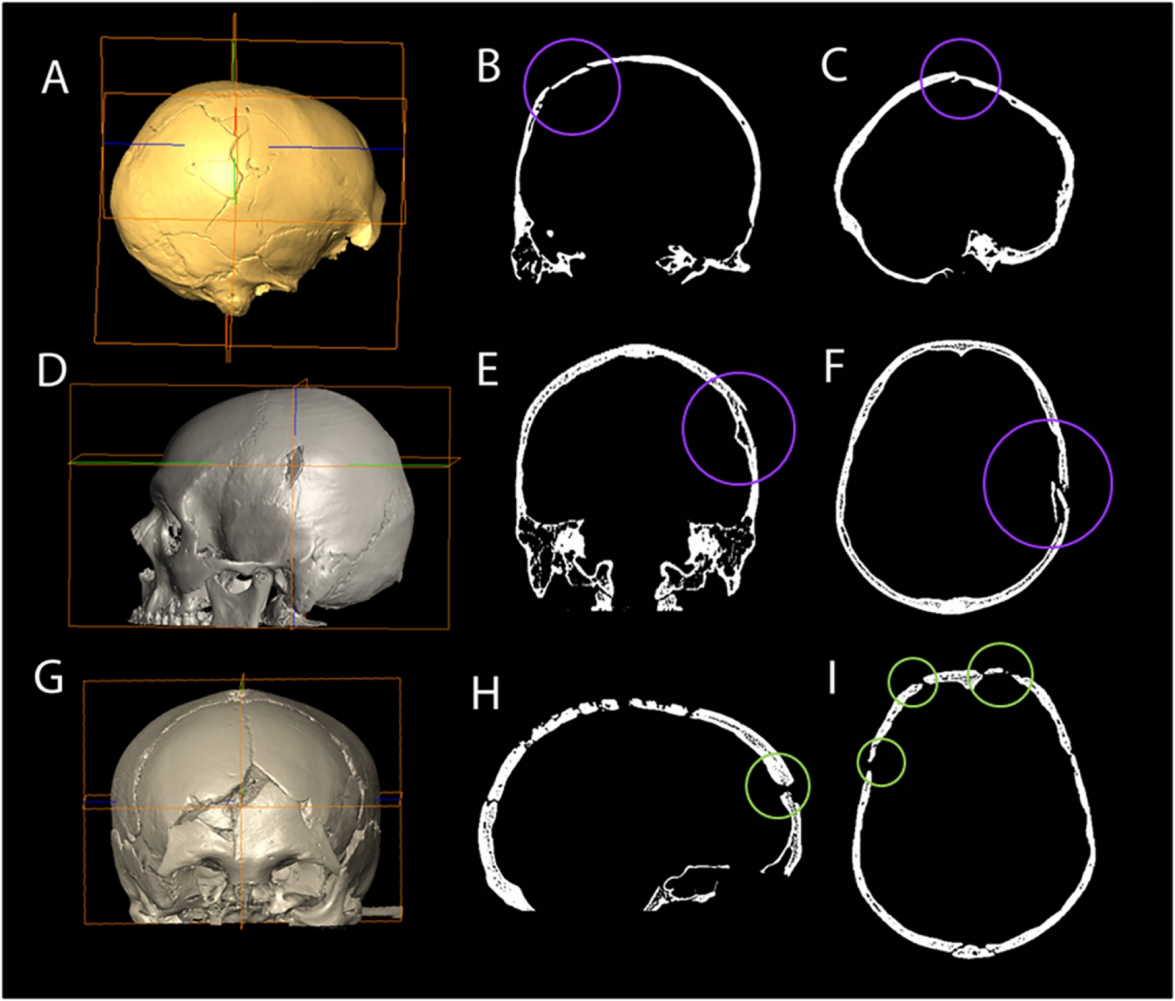
A-C: Cioclovina, D-F: modern cranium with perimortem trauma (data obtained from the archives of the Department of Radiology, Heraklion University Hospital, Crete, Greece with permission), G-I: archaeological cranium with post-mortem damage (medieval specimen from the Ballumbie Collection, Department of Archaeology, University of Edinburgh, Scotland). Note the plastic deformation of the perimortem fractures in the 2-dimensional figures (B, C, E and F) compared to the sharp edges at right angles to the bone surface indicating post-mortem damage (H and I).

**Fig 3:**
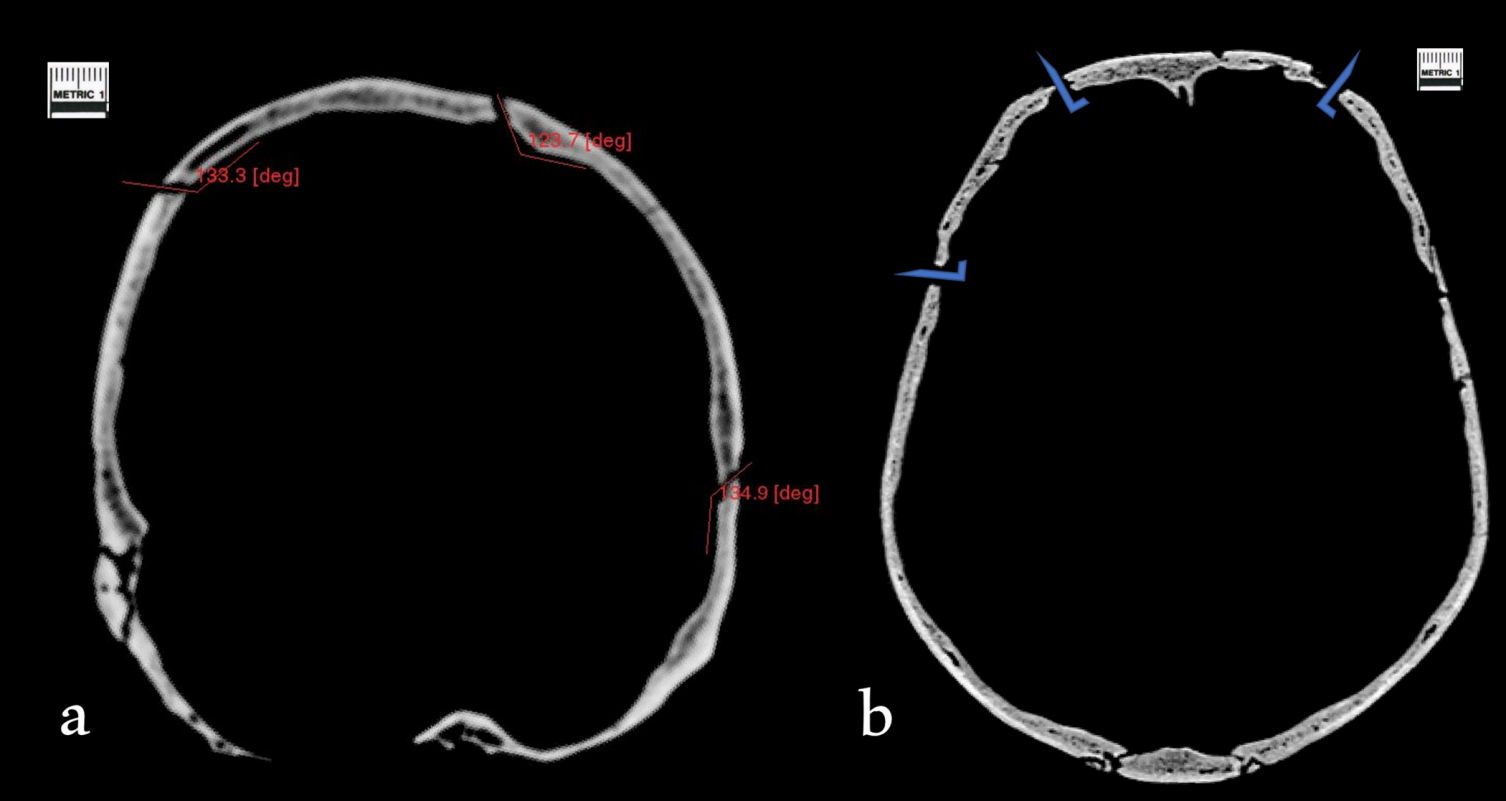
a) Coronal section of a) Cioclovina showing obtuse fracture angles (red lines) consistent with perimortem trauma and b) the Ballumbie skull showing right angle fractures (blue markers) consistent with postmortem damage.

The injuries of Cioclovina present several characteristics of perimortem trauma, including plastic deformation, bone flakes, sharp edge morphology, acute or obtuse fracture angles (Figs 2 and 3) and lack of healing (Table 1).

**Fig 4:**
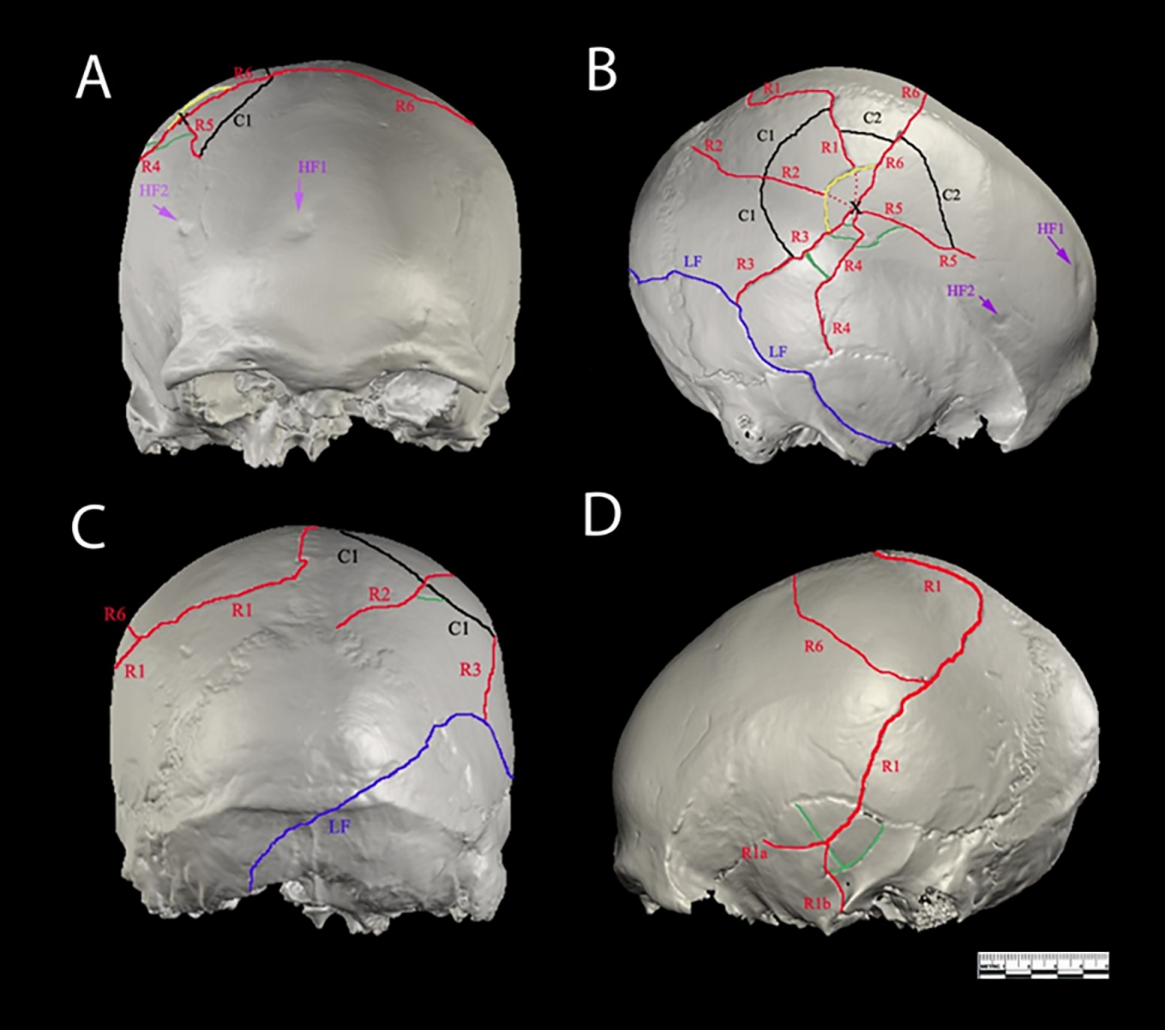
Perimortem fractures LF (in blue) and DF (in red yellow and black) are illustrated in Frontal (A), Occipital (C) Lateral (B,D) views. The right parietal bone of Cioclovina calvaria exhibits a central depressive fracture radiating to the right temporal, occipital and left parietal bones (Fig 2A). The centre of the depression fracture is located on the right parietal bone, approximately 2 cm superiorly to the inferior temporal line. There is an inward displacement of a bow-like bony fragment (r = 35.2 mm). Six linear fractures (R1-R6), radiating from the centre of impact are observed, as well as two main concentric fractures (C1-C2) perpendicular to the radiating fractures (Fig 2B). The first radial fracture (R1) extends superiorly to the centre of impact up to the sagittal suture. There it changes course following the suture towards lambda for about 31 mm. At a distance of approximately 29.5 mm from lambda it turns 90 degrees to the direction of the left parietal bone, crosses the right superior and inferior temporal line and moves slightly posteriorly and downwards to the left parietal squama. It crosses the left squamosal suture in a distance of 40mm from the superior border of the left external acoustic meatus. At that point gives rise to 2 smaller fracture lines (R1a, R1b) which extend to the temporal squama. R1a stops once it meets the temporal suture and R1b reaches the cranial base. R2 starts at the middle of the arc produced by the inward displacement of the central fragment and moves posterior to the right parietal bone stopping at the right lambdoid suture. R3 starts in the middle of the parietal bone, moves to the anterior part of the bone and stops at the edge of a linear fracture (LF). LF starts at the middle left of the inferior nuchal line, moves diagonally accross the tuberculum linearum, crosses the right lamdoid suture, moves slightly inferiorly and anteriorly, crossing the right squamosal suture and the right temporal bone and ends at the inferior part of the right lateral wing of the sphenoid bone (B and C). R4 begins at the impact point slightly posteriorly and then turns inferiorly and parallel to R3, crosses the right inferior temporal line, runs the right parietal bone, and ends at the right squamosal suture about 35.1 mm above the superior border of the right external acoustic meatus. R5 starts from the impact point directly posteriorly to the parietal bone. It turns slightly inferiorly when crossing the inferior temporal line and ends at the right coronal suture. R6 starts at the impact point, moves slightly posteriorly and inferiorly and crosses almost perpendicularly the sagittal suture at a distance of approximately 30.6 mm from bregma. After running approximately 29.5 mm on the posterior part of the left parietal bone, R6 turns 144 degrees anteriorly and inferiorly for approx. 43.9 mm. At the level of the left superior temporal line it turns 131 degrees anteriorly and stops at the R2 fracture line.

Visual inspection of both external and internal surfaces of the cranium using CT imaging revealed two types of perimortem injuries: a depressed fracture (hereafter DF) on the right parietal bone radiating to the right temporal, occipital and left parietal bones; and a linear fracture (hereafter LF) extending from the occipital to the right sphenoid bone. The depressed fracture consists of 6 radial fractures (R1-R6). R1 meets the sagittal suture, follows it towards lambda and then continuous to the left parietal bone and splits into two branches in the left temporal bone. R2, 4 and 5 stop when they meet suture lines, while R6 crosses the sagittal suture and stops when it meets R1 and R3, which stops at the LF (Fig 4). This means that R1 was formed before R6 and LF before the R3 fracture.

Cioclovina shows no signs of healing in either the DF or the LF, ruling out the possibility of antemortem injury. The DF is a typical depressed fracture with evident plastic deformation, inward displacement of a bony fragment still attached to the cranium, and a number of concentric and radiating fractures.

**Fig 5:**
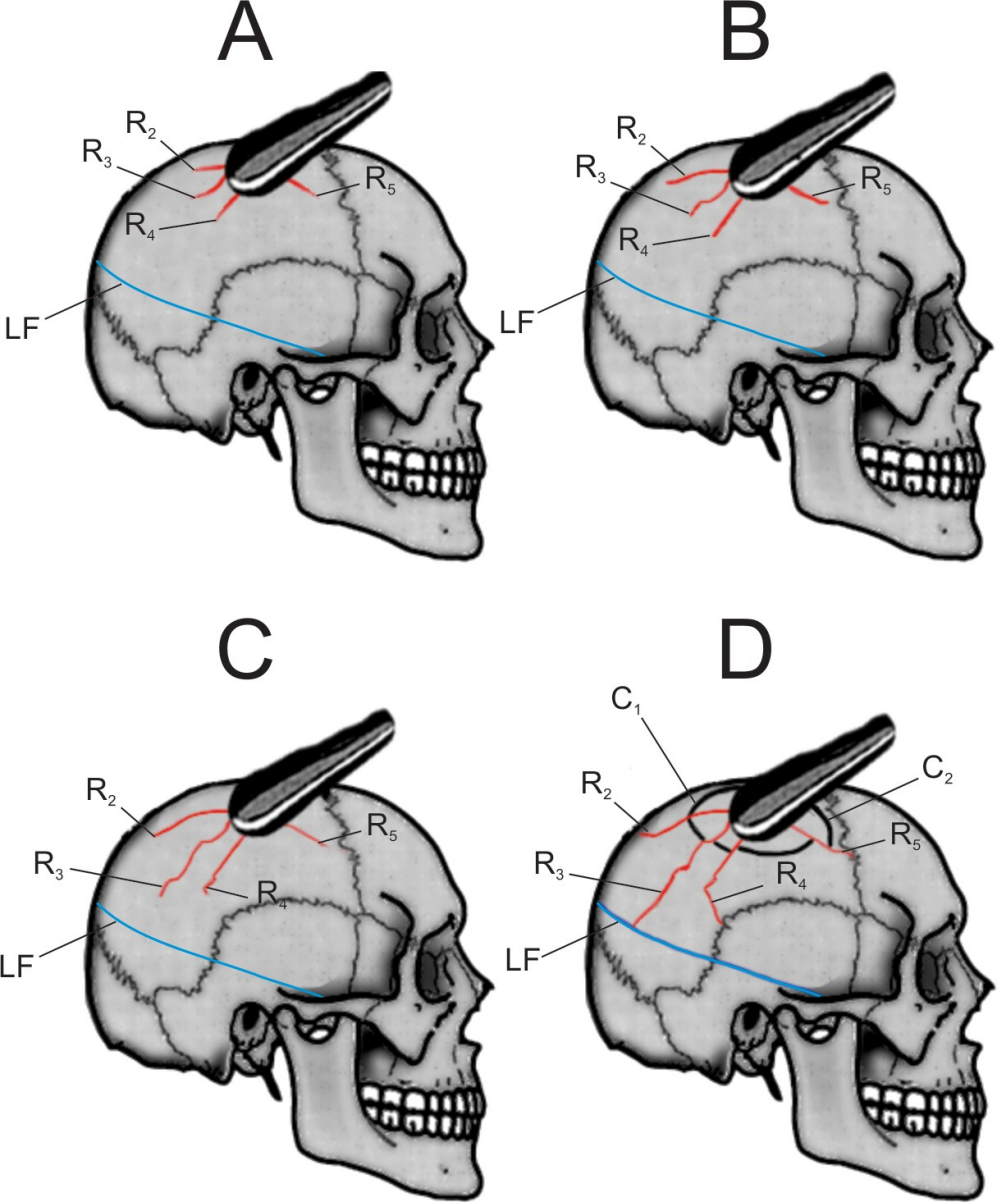
Mechanism of blunt force trauma on the vault A) Low velocity impact on the skull causing fracture formation at the impact point due to initial inbending of the cranial vault with peripheral outbending; inward displacement of the bony fragment due to plastic deformation; small fragments remaining in place suggest that the impact took place while soft tissue was present. B) Radiating fractures in the area of outbending which start at one or more points distant to the impact site, progressing both towards to the impact point and in the opposite direction (away from it); C) The radiating fractures stop when they meet the sutures (e.g. R1). D) Formation of concentric fractures forming perpendicular to the radiating fractures. Image credit: Iakovos Ouranos.

The endocranial aspect of the parietal bone shows one of the radiating fractures following the canal of a branch of the posterior middle meningeal vessel (S1 Fig), consistent with perimortem trauma caused by a blow to the head as illustrated in **Fig 5**.

The depressed and inwardly displaced cranial fragment has a semicircular shape with a radius of approximately 35.2 mm. This shape could only be produced by a rounded object, such as a club [14]. The direction of the inward displacement (arc towards the back) suggests that the trajectory of the blow was antero-posterior and supero-inferior. The rounded edge and slanted direction of the blow also make an accidental injury from cave roof fall debris unlikely to have caused this injury: cave roof debris is expected to be angular and not rounded, and to fall directly overhead rather than in an anterosuperior to posteroinferior direction. The result would have been a comminuted fracture with the cranial fragment entering the cranial cavity instead of an inward displaced fragment (see Methods).

The Cioclovina LF is not crossed by, and is the limit of, the DF radiating fracture R3 (Fig 4). Therefore the LF was sustained before the DF (Methods), and the question arises whether it could have also been produced by a blow. Linear fractures are not associated with a particular type of injury. Although this fracture is found below the HBL, the vast majority of cranial lesions inflicted with baseball bats, a club-like instrument similar to that which likely caused the Cioclovina DF, result in cranial vault and base linear fractures[27]. A similar pattern of linear fracture to the neuro- and basicranium as that shown by Cioclovina was found in historical victims of execution by blow to the back of the head with a club[28]. Therefore, while a fall cannot be excluded as a possible cause for the Cioclovina LF, a blow from the same type of object that caused the DF is a likely cause.

### Blunt force trauma simulations

**Fig 6:**
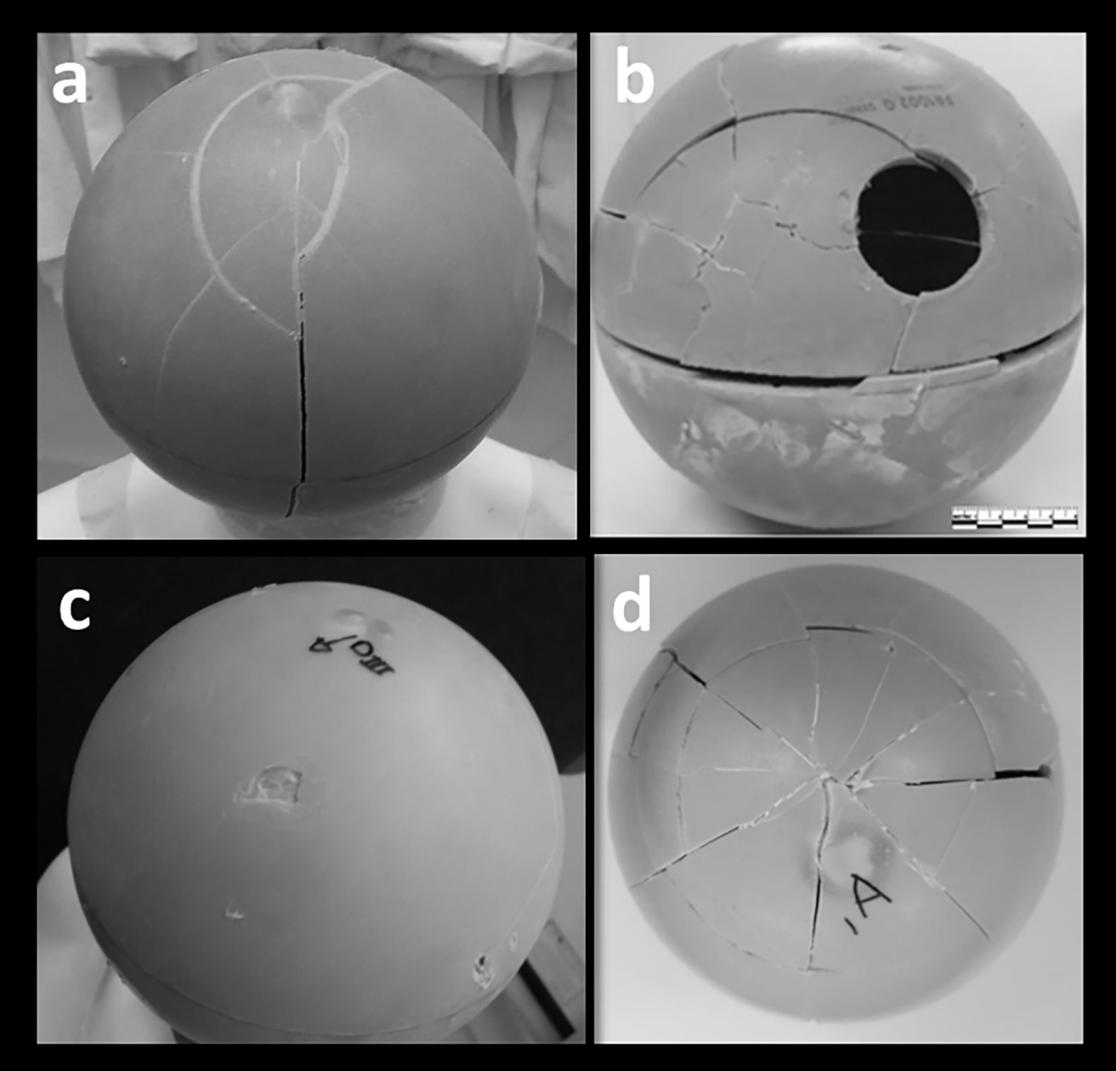
a) Strike with a wooden baseball bat on the vault b) Strike with a wooden baseball bat near the foramen magnum c) strike with a volcanic rock d) Fall from 10 m.

Artificial bone spheres filled with ballistic gelatin to simulate the human head were employed to test different scenarios for the inflicted injuries. The results of the simulations are summarised in Table 2. The main objective was to establish the most likely sequence of events that led to Cioclovina’s death by comparing its facture patterns with the ones observed under controlled experiments (Fig 6). The Cioclovina DF pattern strongly resembles a depressed fracture caused by a round club-like object on a free-standing head (Fig 6B and 6C). The rock created a small puncture fracture (Fig 6D) inconsistent with DF, thus this scenario was rejected. Fig 6E is the result of a free fall from 3 m published by Thali et al.[29] and shown here with permission (No Copyright 4551441316568 (2019), with permission from Elsevier) while Fig 6F is the result of a fall from 10 m. Both these last scenarios result in different patterns than observed in the Cioclovina DF.

**Table 2:** Summary of the BFT simulations.

	Position	Round	Strike N	Wooden Baseball Bat	Rock	Direction
**Fall 3m**	Vault	Data from Thali et al. 2002				
**Fall 10m**		Round 1	Extreme fragmentation			
		Round 2	Extreme fragmentation			
**On a Hard Surface**		Round 3	Strike 1	Linear		Diagonal
			Strike 2	Depressed		
		Round 4	Strike 1		Linear	
		Round 5	Strike 1	Linear		Vertical
			Strike 2	Depressed		
		Round 6	Strike 1		Hairline	
	Neck	Round 7	Strike 1	Depressed		
		Round 8	Strike 1	Depressed		
**Resistance Free**	Vault	Round 9	Strike 1	Depressed		Diagonal
			Strike 2	Depressed		
		Round 10	Strike 1		Puncture	
		Round 11	Strike 1	Depressed		Vertical
			Strike 2	Depressed		
		Round 12	Strike 1		Puncture	

**Fig 7:**
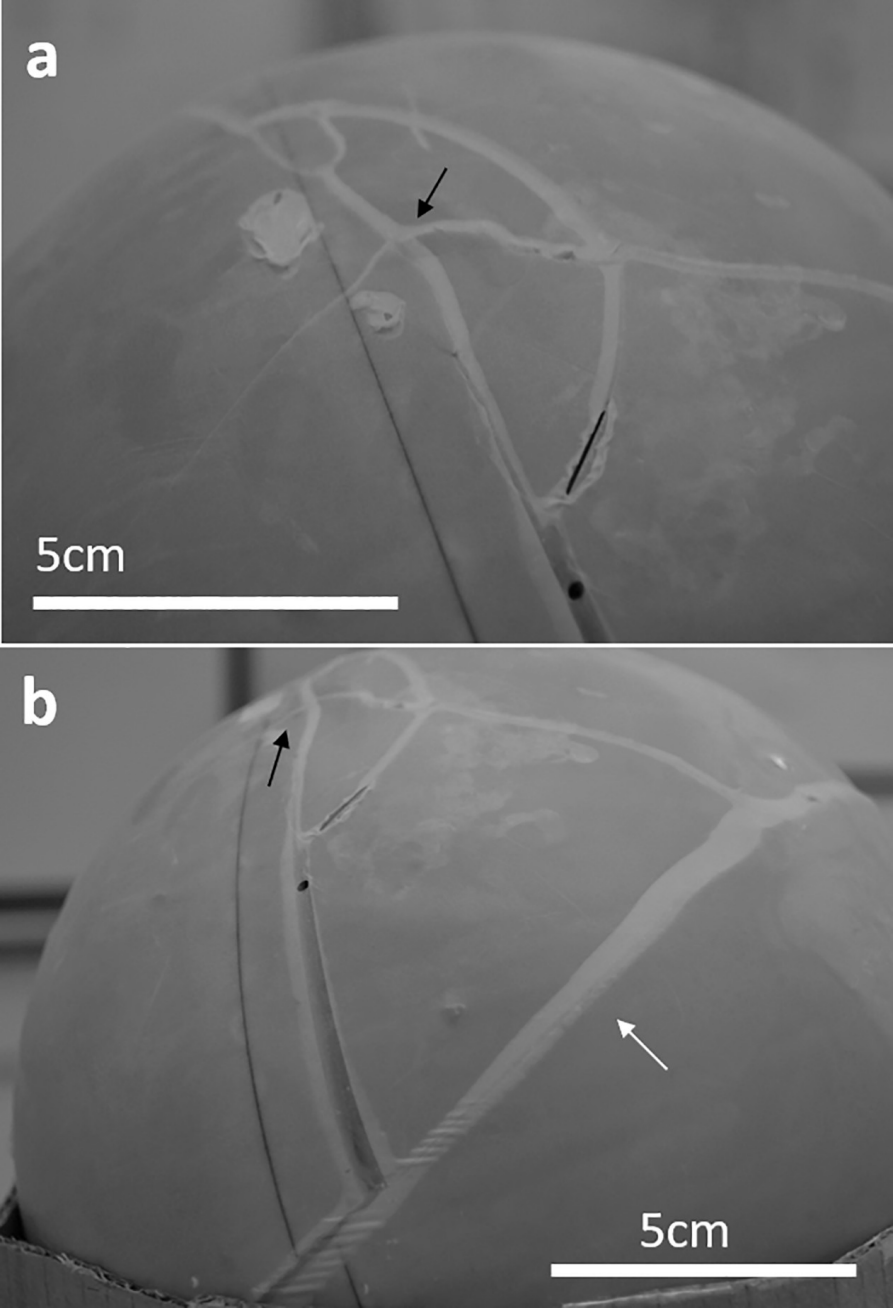
Two strikes with a wooden Baseball bat on a head against a solid surface result in a linear fracture after the first blow (white arrow) and a depressed fracture after the second blow (black arrow).

When the head was free to move both strikes resulted in depressed fractures, while when the head was against a solid surface the first fracture was linear and the second was depressed (Fig 7). This last scenario is consistent with the combination of LR and DF seen in Cioclovina. This leaves open the possibility of both injuries to have emerged as a result of two consecutive blows from the same object. The lack of healing of the DF strongly indicates that it was the fatal injury—although we cannot rule out the possibility of additional injuries on the rest of the body, which is not preserved in the fossil record.

## Discussion

Violent conflict, from personal dispute to group conflicts and war, is common throughout human history. While more recent events are registered through historical documents, skeletal analysis is the only way of assessing evidence for such behaviour in prehistory. Because the reaction of bone to external force is predictable, the observation of fracture patterns allows forensic scientists to deduce the mechanism and circumstances of the inflicted trauma [17]. Our work comprises a thorough forensic analysis of an Upper Paleolithic specimen presenting fatal blunt injuries provoked by a human agent inflicted more than 30.000 years ago. Forensic interpretation is supported by a series of experiments using artificial bone (SYNBONE) spheres that tested several possible scenarios resulting in this fatal trauma. These proxies have been used successfully to reconstruct the circumstances of fatal blunt force injuries in a Neolithic skull [30] as well as to conduct ballistic simulations[31,32].

The analysis of the Cioclovina calvaria revealed at least two perimortem fractures in the occipital and parietal bones, clearly lacking any signs of healing. The DF is a typical depressed fracture with evident plastic deformation, inward displacement of a bony fragment still attached to the cranium, and a number of concentric and radiating fractures. This is a textbook pattern of an injury induced by a blow with a round, bat-like object, as described by several authors[14,15] and confirmed with our SYNBONE experiments (Fig 5B and 5C). The direction of the inward displacement (arc towards the back) suggests that the trajectory of the blow was anterior-posterior and superior-inferior. The pattern and position of DF suggests a blow from an individual in a face-to-face confrontation with the victim. A scenario in which Cioclovina would have received the blow in a kneeling position cannot be excluded. However, in such a case, the head would be at a significantly lower level than a standing individual; thus a blow would cause an impact to the top of the head and not laterally–assuming that the head was in anatomical position and not moving at the time of the impact. Furthermore, the characteristics of the trauma pattern place the blunt object in the left hand of the perpetrator, since the injury is on the right parietal bone, although the possibility of holding the object with both hands cannot be dismissed. Right-handed opponents often strike the left side of their opposition’s skull when facing them[22,33]. This observation was also made in several instances during the SYNBONE experiments.

The radiating fracture R3 (Fig 3), which is part of the DF, stops its course when it meets the LF. Therefore, the LF precedes the DF, and the question arises whether it could have also been produced by a blow. It has been suggested that linear fractures are associated with accidents[15,24] especially when they are located at or under the Hat Brim Line[15], as is the Cioclovina LF. However more recent studies have questioned the validity of this rule[25,28] suggesting that assault cannot be excluded only on the basis of the location of the linear fracture. Indeed, the vast majority of cranial lesions inflicted with baseball bats, a club-like instrument similar to that which likely caused the Cioclovina DF, result in cranial vault and base linear fractures[34]. A similar pattern of linear fracture to the neuro- and basicranium as the one shown by Cioclovina was documented in historical victims of execution by blow to the back of the head with a club[28]. This is also consistent with our SYNBONE experiments (strike with wooden baseball bat near the foramen magnum, Fig 4C). Furthermore, the SYNBONE experiments resulted in linear fractures after striking the spheres, which were fixed against a solid surface simulating assault on a victim with the head against the ground or the wall (Fig 5A).

In contrast, the simulations of falls from 10 m both resulted in shattering of the spheres in multiple pieces (Fig 4E). A fall from 3 m height, as simulated by Thali and colleagues[29] using artificial bone, also resulted in a pattern which is highly inconsistent with the DF on Cioclovina (see Fig 4E). Recent studies report that the occurrence of cranial base fractures (linear fracture, linear fracture of the foramen magnum or occipital condyles) were 10 times more likely to occur in falls from 1.5–3 m rather than from falls of <1.5 m[35]. This has been attributed to the fact that falls from lower than 1,5 m or higher than 3 m do not result in people landing on their head. Subsequently, a fall from a small height 1,5–3 m cannot be excluded as a possible cause for the Cioclovina LF, while a blow with the same type of object that caused the DF is also a likely cause.

The combined evidence produced by the application of clear-cut forensic criteria and experimental data indicates that the pattern of the Cioclovina fractures is inconsistent with post-mortem damage, or with injuries sustained due to a fall alone, or with an accidental injury due to roof fall debris. Rather, they were sustained from multiple blows to the head with a club-like instrument, or from a combination of a fall and a blow to the head. The lack of any signs of healing associated with these fractures indicates that the Cioclovina individual did not survive these lesions. It is not clear whether the LF alone could have caused his death. However, a depressed fracture of the extent and magnitude of the Cioclovina DF would have caused fatal brain injuries resulting in a quick demise. The location of this lesion suggests that it was inflicted by a blow from a likely left-handed perpetrator facing the victim. This may have been a result of a one to one conflict or murder by one or more perpetrators. Severe interpersonal conflict leading to death is therefore the hypothesis that is best supported by our findings.

Forensic evidence suggests that this early modern European suffered a violent death caused intentionally by another human. Signs of interpersonal violence with a variety of weapons are very common in antiquity [30,36–38]. Previous work has showed a level of interpersonal aggression among Neanderthals[39,40] and even earlier hominins[41–43], and early Holocene hunter-gatherers[44] (although some of this evidence has been questioned[45]). A case of possible interpersonal violence among early Upper Paleolithic *Homo sapiens* has been documented for the individual Sunghir 1[42], who shows a fatal perimortem sharp trauma in the first thoracic vertebra. However, this lesion is not considered a clear-cut case of lethal interpersonal violence, as the possibility of a hunting accident could not be dismissed. In contrast, the fatal injuries of the Middle Pleistocene fossil (Cranium 17) from the *Sima de los Huesos* were identified as BFT injuries caused by the same shape object on the frontal bone, and thus represent indisputable evidence of interpersonal conflict[41]. Similarly, the injuries in Cioclovina constitute clearly documented perimortem injuries that can be securely attributed to human intentional actions, as the Cioclovina DF could not represent anything but a fatal blow. This evidence therefore testifies that homicide was among the behavioral repertoire of Aurignacian modern humans *ca*. 33 thousand years ago. Such behavior is perhaps not surprising given its prevalence in later periods and the mounting evidence for violence among early human groups[33,40–42,46], as well as non-human primates[47].

## Supporting information

S1 Fig A. Linear fracture trajectory in Cioclovina ectocranial surface. B. Linear fracture trajectory (blue) and posterior branch of the middle meningeal vessels trajectory (red) in Cioclovina endocranial surface. C. Virtual reconstruction of Cioclovina endocast with memingeal and sinus configurations. Note the part of the posterior branch of the middle meningeal vessels corresponding to the imprint crossed by the linear fracture (red highlighted with white).
(TIF)

S1 Online Material Detailed description of the experimental trauma simulation with artificial bone spheres. (DOCX)

## References

[1] Rainer F, Simionescu I. Sur le premier crâne d’homme Paléolithique trouvé en Roumanie. Analele Acad Rom Memoriile Secţiunii Ştiinţifice, Ser III. 1942;17: 489–503.

[2] Soficaru A, Petrea C, Dobos A, Trinkaus E. The Human Cranium from the Peştera Cioclovina Uscată, Romania: Context, Age, Taphonomy, Morphology, and Paleopathology. Cur Anthr. 2007;48: 611–619.

[3] Harvati K, Gunz P, Grigorescu D. Cioclovina (Romania): affinities of an early modern European. J Hum Evol. Academic Press; 2007;53: 732–746. 10.1016/j.jhevol.2007.09.009 18001819

[4] Kranioti EF, Holloway R, Senck S, Ciprut T, Grigorescu D, Harvati K. Virtual Assessment of the Endocranial Morphology of the Early Modern European Fossil Calvaria From Cioclovina, Romania. Anat Rec (Hoboken). 2011;294: 1083–1092. 10.1002/ar.21420 21634023

[5] Uhl A, Reyes-Centeno H, Grigorescu D, Kranioti EF, Harvati K. Inner ear morphology of the cioclovina early modern European calvaria from Romania. Am J Phys Anthropol. 2016;160 10.1002/ajpa.22938 26806095

[6] Fu Q, Posth C, Hajdinjak M, Petr M, Mallick S, Fernandes D, et al The genetic history of Ice Age Europe. Nature. Nature Publishing Group; 2016;534: 200–205. 10.1038/nature17993 27135931 PMC4943878

[7] Ullrich H. Artifizielle Veränderungen am Jungpaläolithischen Schädel von Cioclovina (S. R. Rumänien). Annu Romaine d’Anthropologie. 1979;16: 3–12.

[8] Işcan MY, Steyn M. Bone Pathology and Antemortem Trauma. The Human Skeleton in Forensic Medicine. 3rd ed Springfield: Charles C. Thomas; 2013 pp. 291–316. 10.1016/B978-0-12-382165-2.00014-3

[9] Kranioti E. Forensic investigation of cranial injuries due to blunt force trauma: current best practice. Res Reports Forensic Med Sci. 2015;5: 25–37. 10.2147/RRFMS.S70423

[10] Sauer N. The timing of injuries and manner of death: distinguishing among antemortem, perimortem and post-mortem trauma In: Reichs K, editor. Forensic Osteology: Advances in the Identification of Human Remains. 1st ed Springfield, Illinois: CC Thomas; 1998 pp. 321–332.

[11] Barbian L, Sledzik P. Healing following cranial trauma. J Forensic Sci. 2008;53: 263–268. 10.1111/j.1556-4029.2007.00651.x 18298494

[12] Moritz A. Pathology of trauma. 2nd ed Philadelphia: Lea & Febiger; 1954.

[13] Fleming-Farrell D, Michailidis K, Karantanas A, Roberts N, Kranioti EF. Forensic Anthropology Population Data Virtual assessment of perimortem and postmortem blunt force cranial trauma. 10.1016/j.forsciint.2013.03.032 23601150

[14] Berrymann H, Symes SA. Recognizing gunshot and blunt cranial trauma through fracture interpretation In: Reichs K, editor. Forensic Osteology: Advances in the Identification of Human Remains. 1st ed Springfield, Illinois: CC Thomas; 1998 pp. 333–352.

[15] DiMaio VJ, DiMaio D. Forensic Pathology In: V DJD, editor. Forensic Pathology. 2nd ed CRC Press, LLC; 2001.

[16] Blau S. How traumatic: a review of the role of the forensic anthropologist in the examination and interpretation of skeletal trauma. Aust J Forensic Sci. Taylor & Francis; 2017;0618: 1–20. 10.1080/00450618.2016.1153715

[17] Berryman HE, Berryman JF, Saul TB. Bone trauma analysis in a forensic setting: Theoretical basis and a practical approach for evaluation. In: Boyd C, Boyd D, editors. Forensic Anthropology: Theoretical Framework and Scientific Basis Chichester, UK: John Wiley & Sons Ltd; 2018 pp. 213–234.

[18] Wescott DJ. Biomechanics of bone trauma. In: Siegel J, Saukko P, Houck M, editors. Encyclopedia of Forensic Sciences New York: Elvesier; 2013 pp. 253–277.

[19] Dirkmaat DC, Cabo LL, Ousley SD, Symes SA. New perspectives in forensic anthropology. Am J Phys Anthropol. 2008;137: 33–52. 10.1002/ajpa.20948 19003882

[20] Dirkmaat DC, Cabo LL, Ousley SD, Symes SA. New perspectives in forensic anthropology. Am J Phys Anthropol. 2008;137: 33–52. 10.1002/ajpa.20948 19003882

[21] Symes S, L’Abbé E, Chapman E, Wolff I, Dirkmaat DC. Interpreting traumatic injury to bone in medicolegal investigations. In: Dirkmaat DC, editor. A Companion to Forensic Anthropology. 1st ed Chichester, UK: John Wiley & Sons, Ltd; 2012 pp. 340–389.

[22] Lovell NC. Trauma Analysis in Paleopathology. Yrbk Phys Anthr. 1997;40: 139–170. 10.1002/(SICI)1096-8644(1997)25+<139::AID-AJPA6>3.0.CO;2

[23] Calce SE, Rogers TL. Taphonomic changes to blunt force trauma: A preliminary study. J Forensic Sci. 2007;52: 519–527. 10.1111/j.1556-4029.2007.00405.x 17397504

[24] Kremer C, Racette S, Dionne CA, Sauvageau A. Discrimination of falls and blows in blunt head trauma: Systematic study of the hat brim line rule in relation to skull fractures. J Forensic Sci. 2008;53: 716–719. 10.1111/j.1556-4029.2008.00725.x 18471221

[25] Fracasso T, Schmidt S, Schmeling A. Commentary on: Kremer C, Racette S, Dionne CA, Sauvageau A. Discrimination of falls and blows in blunt head trauma: Systematic study of the hat brim rule in relation to skull fractures. J Forensic Sci 2008 5; 53(3):716–9. J Forensic Sci. 2011;56: 1662. 10.1111/j.1556-4029.2008.00725.x 22040041

[26] Madea B, Staak M. Determination of the sequence of gunshot wounds of the skull. J Forensic Sci Soc. 1988;28: 321–328. 3068330 10.1016/s0015-7368(88)72858-3

[27] Dujovny M, Onyekachi I, Perez-Arjona E. Baseball bats: a silent weapon. Neurol Res. 2009;31: 1005–11. 10.1179/174313209X385716 19203443

[28] Taala SC, Berg GE, Haden K. Blunt force cranial trauma in the Cambodian killing fields. J Forensic Sci. 2006;51: 996–1001. 10.1111/j.1556-4029.2006.00219.x 17018075

[29] Thali MJ, Kneubuehl BP, Dirnhofer R. A “skin-skull-brain model” for the biomechanical reconstruction of blunt forces to the human head. Forensic Sci Int. 2002;125: 195–200. 10.1016/S0379-0738(01)00639-9 11909663

[30] Dyer M, Fibiger L. Understanding blunt force trauma and violence in Neolithic Europe: the first experiments using a skin-skull-brain model and the Thames Beater. Antiquity. 2017/12/06. Cambridge University Press; 2017;91: 1515–1528. 10.15184/aqy.2017.189

[31] Smith MJ, James S, Pover T, Ball N, Barnetson V, Foster B, et al Fantastic plastic? Experimental evaluation of polyurethane bone substitutes as proxies for human bone in trauma simulations. Leg Med (Tokyo). Ireland; 2015;17: 427–435. 10.1016/j.legalmed.2015.06.007 26130519

[32] Taylor SC, Kranioti EF. Cranial trauma in handgun executions: Experimental data using polyurethane proxies. 2018; 10.1016/j.forsciint.2017.11.032 29202338

[33] Walker PL. A Bioarchaeological Perspective on the History of Violence. 2001;30: 573–576.

[34] Dujovny M, Onyekachi I, Perez-Arjona E. Baseball bats: a silent weapon. Neurol Res. 2009;31: 1005–11. 10.1179/174313209X385716 19203443

[35] Rowbotham SK, Blau S, Hislop-Jambrich J, Francis V. Skeletal Trauma Resulting From Fatal Low (≤3 m) Free Falls: An Analysis of Fracture Patterns and Morphologies. J Forensic Sci. 2017; 1–11. 10.1111/1556-4029.13701 29193109

[36] Messina AD, Carotenuto G, Miccichè R, Sìneo L. Fatal cranial injury in an individual from Messina (Sicily) during the times of the Roman Empire. J Forensic Leg Med. Elsevier Ltd; 2013;20: 1018–1023. 10.1016/j.jflm.2013.09.023 24237811

[37] Pasini A, Gualdi-Russo E, Scianò F, Thun Hohenstein U. Violence in the Early Bronze Age. Diagnosis of skull lesions using anthropological, taphonomic and scanning electron microscopy techniques. Forensic Sci Med Pathol. Forensic Science, Medicine and Pathology; 2018; 10.1007/s12024-018-0054-z 30547355

[38] Schulting R, Fibiger L. Skeletal evidence for interpersonal violence in Neolithic Europe Schulting R, Fibiger L, editors. Oxford: Oxford Scholarship; 2012 10.1093/acprof:osobl/9780199573066.003.0001

[39] Zollikofer CPE, Ponce de Leon MS, Vandermeersch B, Leveque F. Evidence for interpersonal violence in the St. Cesaire Neanderthal. Proc Natl Acad Sci. 2002;99: 6444–6448. 10.1073/pnas.082111899 11972028 PMC122968

[40] Churchill SE, Franciscus RG, McKean-Peraza HA, Daniel JA, Warren BR. Shanidar 3 Neandertal rib puncture wound and paleolithic weaponry. J Hum Evol. 2009;57: 163–178. 10.1016/j.jhevol.2009.05.010 19615713

[41] Sala N, Arsuaga JL, Pantoja-Pérez A, Pablos A, Martínez I, Quam RM, et al Lethal interpersonal violence in the middle pleistocene. PLoS One. 2015;10: 1–12. 10.1371/journal.pone.0126589 26018668 PMC4446311

[42] Trinkaus E, Buzhilova AP. The death and burial of sunghir 1. Int J Osteoarchaeol. 2012;22: 655–666. 10.1002/oa.1227

[43] L’Abbé EN, Symes SA, Pokines JT, Cabo LL, Stull KE, Kuo S, et al Evidence of fatal skeletal injuries on Malapa Hominins 1 and 2. Sci Rep. The Author(s); 2015;5: 15120 Available: 10.1038/srep15120 26459912 PMC4602312

[44] Lahr MM, Rivera F, Power RK, Mounier A, Copsey B, Crivellaro F, et al Inter-group violence among early Holocene hunter-gatherers of West Turkana, Kenya. Nature. Nature Publishing Group; 2016;529: 394–398. 10.1038/nature16477 26791728

[45] Stojanowski C, Seidel A, Fulginiti L, Johnson K, Buikstra J. Contesting the massacre at Nataruk. Nature. 2016;539. 10.1038/nature19778 27882979

[46] Zollikofer CPE, Ponce de Leon MS, Vandermeersch B, Leveque F. Evidence for interpersonal violence in the St. Cesaire Neanderthal. Proc Natl Acad Sci. 2002;99: 6444–6448. 10.1073/pnas.082111899 11972028 PMC122968

[47] Shimada M, Uno T, Nakagawa N, Fujita S, Izawa K. Case study of a one-sided attack by multiple troop members on a nontroop adolescent male and the death of japanese macaques (Macaca fuscata). Aggress Behav. 2009;35: 334–341. 10.1002/ab.20308 19431186

