# Supplementary figures and images for: State of the art forensic techniques reveal evidence of interpersonal violence ca. 30,000 years ago
Source: PLoS One. 2019 Jul 3;14(7):e0216718. doi: 10.1371/journal.pone.0216718 (PMC6608943; doi:10.1371/journal.pone.0216718)

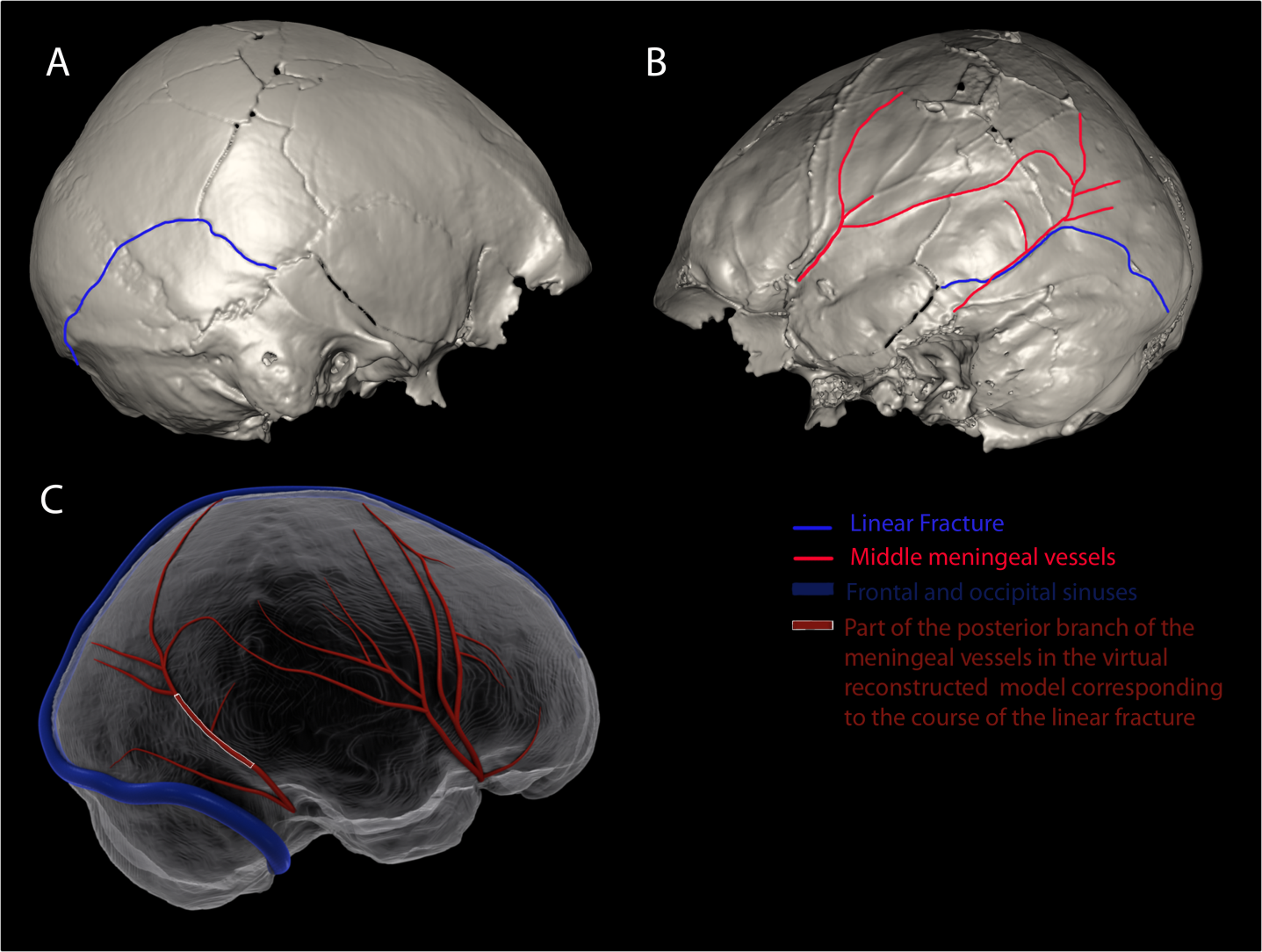

Supplement: S1 Fig — A. Linear fracture trajectory in Cioclovina ectocranial surface. B. Linear fracture trajectory (blue) and posterior branch of the middle meningeal vessels trajectory (red) in Cioclovina endocranial surface. C. Virtual reconstruction of Cioclovina endocast with memingeal and sinus configurations. Note the part of the posterior branch of the middle meningeal vessels corresponding to the imprint crossed by the linear fracture (red highlighted with white). (TIF) [file pone.0216718.s001.tif]
