# Supplementary material for: State of the art forensic techniques reveal evidence of interpersonal violence ca. 30,000 years ago
Source: PLoS One. 2019 Jul 3;14(7):e0216718. doi: 10.1371/journal.pone.0216718 (PMC6608943; doi:10.1371/journal.pone.0216718)
Supplement: S1 Online Material — (DOCX) [file pone.0216718.s002.docx]

Supplementary material

**Gelatine Preparation**

The protocol followed for the gelatine preparation was the one developed by Fackler and Malinowski [1-2] and also followed by Sellier and Kneubuehl [3]. After the first batch was prepared according to the article, however, it was found to be too substantial and was creating more waste than acceptable. It was then decided to take one eighth of all the components out. Presented below is the revised procedure, adjusted for less wastage.

For 10% ballistic gel:

1. 315g of gelatine powder was poured into 2.835l of boiled cold water (7-10℃) for each sphere.
2. The gelatine powder was gently mixed into the cold water until evenly distributed and completely submerged.
3. The mixture then needs to be refrigerated for 2h.
4. The solution is then heated in a hot water bath and slowly stirred until entirely homogenous and clear. During that phase, the temperature of the gelatine blend must not exceed 40℃.
5. The solution can be then poured into the sphere which need be refrigerated to set for at least 48 hours before use.

Once the solution is set, the spheres should be used as soon as possible and always kept in the refrigerator to avoid melting until the experiment. Once used, the sphere should be put back in the refrigerator and emptied as quickly as possible to prevent any olfactory nuisance.

To empty the spheres, care should be taken not to damage the fracture inflicted during the experiment and to collect all subsequent pieces and slivers of bone that may have been dislodged during the process. By practice, it was found that the most convenient manner to empty the spheres was to put them in a hot water bath and pour out the gelatine when melted. The rubber coating is then peeled off and the spheres can be kept in any storage room, with no refrigeration or humidity levels control necessary.

**Experimental settings**

**1.Free moving head Scenario**

The setting included a cork ring placed on the neck of a mannequin, to which the sphere was affixed using tape. A set of experiments took place after fixing the mannequin torso on a chair at 1.60 metres height.

**2.Head against a Solid Surface Scenario**

A square metal rigid and immobile surface was placed under the cork ring to simulate a wall or floor.

1. *Simulation of a fall from 10m height (spheres fixed on manikin)*

3m: Thali et al., presented the result of a fall from 3m (Figure 4e)

10m: Spheres S11 and S12 were used to simulate a fall from 10 m height on a solid surface. In both cases the experiment resulted in severe fragmentation of the sphere in several pieces of different shapes and sizes (See example in Figure 4f)

1. *Simulation of a single blow with a volcanic rock (spheres fixed on manikin)*

Stones and rock are similar to clubs, they are extremely easy to come by and therefore have been used as weapons throughout history. The rock is a hard igneous stone. It has irregular shape with a length of 94.8 millimetres, a maximum diameter of 79.5 millimetres and a minimum diameter of 35.9 millimetres. It weighs 148 grams. Spheres S3 and S4 fixed on the manikin were hit with the rock once.

1. *Simulation of a* single and two consecutive blows with a baseball bat on a head free to move (spheres fixed on manikin).

A wooden baseball bat (Louisville Slugger 180 Grand Slam, Ash and the Nike Aero Launch Max 7050) was used for this experiment. The wooden bat is 840mm long and its maximum and minimum diameter is respectively 62.5mm and 23.mm by the handle. It weighs 702 grams. Spheres S5, S6 and S7, S8 were struck once and twice respectively.

1. Simulation of a single and two consecutive blows with a baseball bat on a solid surface

The same wooden bat was used for this round of experiments. Spheres S9, S10 and S11, S12 were struck once and twice respectively.

References

1)Fackler, M.L., Malinowski, J.A., 1985. The Wound Profile: A Visual Method for Quantifying Gunshot Wound Components. J Traum. 25 (6): 522-529.

2)Fackler, M.L., Malinowski, J.A., 1988. Ordnance Gelatine for Ballistic Studies. Detrimental Effect of Excess Heat used in Gelatine Preparation. Am J Foren Med and Path. 9 (3): 218-219.

3)Sellier, K.G., Kneubuehl, B.P., 1994. Wound Ballistics and the Scientific Background. First Edition. Elsevier. Amsterdam.

4) https://www.Synbone1.ch/wEnglish/catalogue/index.php?navanchor=101004. Synbone Company website. 2018. <https://www.synbone1.ch/wEnglish/catalogue/index.php?navanchor=101004>.
